# Development of a clinical algorithm-based scoring system to diagnose smear-negative pulmonary tuberculosis in Sabah, Malaysia using the modified Delphi method

**DOI:** 10.7189/jogh.16.04085

**Published:** 2026-02-20

**Authors:** Chee Kuan Wong, Wai Khew Lee, Roddy Teo, Hema Y Ramamurthy, Jiloris Dony, Chin Hai Teo, Sarah Jane JC Chan, Suhashini Sivasegaran, Yao Long Lew, Ri Hui Lam, Karuthan Chinna, Giri S Rajahram, Timothy William, Yin Chin Chan, Jayakayatri J Nathan, Harish Nair, Harry Campbell, Ee Ming Khoo, Helen R Stagg, AMASSMENT Expert Panel, Chong Kin Liam, Yong Kek Pang, Mat Zuki Mat Jaeb, Nadia Atiya, Kiew Lee Boon, Aikhiang Goon, Bee Kiau Ho, Juliana I Abdul Jalal, Asmah Razali, Zamzurina Abu Bakar, Norlaily Hassan, Haryati Hamzah, Wan Najwa Z Wan Muhamed, Sathya Rao Jogulu, Zaki Zaili, Lalitha Pereirasamy, Maila Mustapha, Zuhanis Abdul Hamid, Narul Aida Salleh, Richard Avoi, Kunji K Kannan, Wan Nurhafizah WA Hamed, Dalyana Hamid

**Affiliations:** 1 Department of Medicine, Faculty of Medicine, Universiti Malaya, Kuala Lumpur, Malaysia; 2 Ministry of Health, Putrajaya, Malaysia; 3 Department of Primary Care Medicine, Faculty of Medicine, Universiti Malaya, Kuala Lumpur, Malaysia; 4 Menzies School of Health Research, Charles Darwin University, Darwin, Australia; 5 UCSI University, Kuala Lumpur, Malaysia; 6 Queen Elizabeth Hospital II, Ministry of Health, Sabah, Malaysia; 7 Subang Jaya Medical Centre, Selangor, Malaysia; 8 Centre for Global Health, Usher Institute, University of Edinburgh, Scotland, UK; 9 Department of Infectious Disease Epidemiology, London School of Hygiene & Tropical Medicine, London, UK

## Abstract

**Background:**

Tuberculosis (TB) remains a major global health threat, particularly in resource-constrained settings where delayed diagnosis of smear-negative pulmonary TB (SNPTB) is common due to limited access to rapid molecular diagnostics. We aimed to develop a clinical algorithm-based scoring system to aid the diagnosis of SNPTB among symptomatic patients in Sabah, Malaysia.

**Methods:**

We conducted a modified Delphi process between January and June 2024 involving three rounds of expert consultation via email to identify key clinical parameters for diagnosing SNPTB, followed by a consensus meeting to finalise the parameters and assign weightings. We then applied the algorithm to a data set of 60 symptomatic smear-negative individuals, of whom 29 were confirmed to be TB and 31 not TB based on culture. We calculated the sensitivity, specificity, positive predictive value (PPV), and negative predictive values (NPV) of the algorithm to obtain a cut-off score for ‘likely TB’ *vs*. ‘unlikely TB’.

**Results:**

Of 27 invited experts, 23 (85.2%) consented to participate in the Delphi process and contributed to the final consensus. Fifty-four parameters were identified in round 1, reduced to 26 in round 2 and 23 in round 3. Following the consensus meeting, we incorporated 21 weighted parameters (scores 1–10) into the final algorithm. The clinical algorithm achieved an area under the receiver operating characteristic curve of 0.88. A cut-off score of 19.5 differentiated ‘likely TB’ from ‘unlikely TB’, yielding a sensitivity of 86.2%, specificity of 77.4%, PPV of 78.1%, and NPV of 85.7%.

**Conclusions:**

This diagnostic clinical algorithm could help doctors practicing in resource-constrained settings to diagnose SNPTB. A next step for research would be the prospective validation of the algorithm.

Tuberculosis (TB) remains a major global public health problem, accounting for an estimated 10.8 million infections and 1.25 million people deaths worldwide in 2023, with over 80% occurring in low- and middle-income countries [1]. There was an upward trend in TB incidence globally following the COVID-19 pandemic, with most new cases occurring in the World Health Organization (WHO) regions of South-East Asia (45%), Africa (24%) and the Western Pacific (17%) [2]. In this context, timely and accurate diagnoses is critical to strengthening TB prevention and care [3].

While the national TB incidence in Malaysia was 78.3 per 100 000 population in 2023, Sabah, a state in the east of the country, saw a 2-fold rate at 160.3 per 100 000 population [4]. Smear-negative pulmonary TB (SNPTB) cases in Sabah constitute about 20% of all cases of pulmonary TB in the state [5]. Although the use of rapid molecular diagnostic tests has been incorporated into the latest Malaysian clinical practice guidelines for SNPTB since 2021, in line with WHO recommendations, their use remains limited in Sabah due to the high costs and limited availability outside tertiary hospitals [6]. Between 2012 and 2018, rapid molecular diagnostic test coverage was only 1.2% of notified TB cases in Sabah [5]; most SNPTB cases in Sabah are diagnosed clinically. A standardised diagnostic algorithm that can be used in the absence of rapid molecular diagnostic tests, benchmarked against GeneXpert and culture, would therefore be highly useful in this setting.

While algorithms for diagnosing SNPTB have already been developed in different contexts, many national programmes have adopted the WHO 2003 clinical algorithm [7], which has not been formally validated and lacks explicit clinical or radiological indicators, limiting its diagnostic accuracy. Indeed, a Cochrane review by Van’t Hoog *et al*. found that combining symptom-based and chest x-ray screening improves diagnostic yield, although sensitivity and specificity vary by region [8]. As a result, some countries have developed their own algorithms or adapted the WHO algorithm to include both clinical and radiographic criteria.

Existing diagnostic algorithms for SNPTB have shown variable performance across different settings, with many models demonstrating reduced accuracy compared with *Mycobacterium tuberculosis* culture and facing challenges in external validation (**Table 1**) [9–17]. Classical and artificial intelligence (AI)-enhanced diagnostic studies for SNPTB report a wide range of performance, from inconsistent sensitivity in symptom-and-CXR algorithms to moderate-high accuracy with neural networks and automated microscopy, and up to radiologist-level performance with deep-learning computer-aided detection and AI-enabled lung ultrasound (**Table 2**) [18–28]. This highlights the substantial advantages of data-driven and multimodal approaches over conventional rule-based methods.

**Table 1:** AI-generated summary of studies evaluating clinical or algorithmic approaches for SNPTB

Study, year	Country	Population	Type of algorithm*	Description of algorithm	Comparator	Diagnostic performance†
Harries *et al*., 2001 [9]	Malawi	Hospital patients with suspected smear-negative PTB	Country-specific (audit of national practice)	Modified national clinical diagnostic algorithm	Routine clinical practice (no formal comparator)	Reported higher accuracy, but unreliable due to poor adherence and documentation; no formal sensitivity/specificity reported
Swai *et al*., 2011 [10]	Tanzania	Patients assessed using National TB and Leprosy Programme algorithm	Country-specific (national programme algorithm)	NTLP symptom-based and radiographic algorithm	Evaluated independently (no external reference)	Sensitivity 38.1%; Specificity 74.5%
Nguyen *et al*., 2012 [11]	Vietnam	HIV-infected individuals with suspected smear-negative TB	Research tool	Symptom-based algorithm; performance reassessed with addition of chest x-ray and CD4 < 200 cells/mm^3^	Sputum culture (single sample)	Symptoms alone: Sensitivity 46%; adding CXR + CD4 improved prediction but remained suboptimal
Kanaya *et al*., 2001 [12]	USA (TPS derivation)	Retrospective cohort of smear-negative TB suspects in low HIV-prevalence setting	Research tool	Tuberculosis Prediction Score (TST+, sputum production, non-TB infiltrates, HIV+, mediastinal lymphadenopathy)	Internal validation only	Numerical sensitivity/ specificity not reported; applicability limited to low HIV-burden settings
Tessema *et al*., 2001 [13]	Ethiopia	Patients attending Addis Ababa TB Centre	Research tool	Weighted clinical and radiological score validated against expert diagnosis	Expert clinical diagnosis (no microbiological reference)	Sensitivity 93%; Specificity 94%; potential circular diagnostic bias noted
Wilkinson *et al*., 2000 [14]	South Africa	Hospitalised adults with suspected TB in high HIV prevalence setting	Country-specific (local clinical algorithm)	Two-course trial-of-antibiotic algorithm (amoxicillin → erythromycin)	Culture-confirmed TB	Sensitivity 89%; Specificity 84%; limited generalisability to outpatient settings
Soto *et al*., 2013 [15]	Peru	Patients in high-incidence, resource-constrained settings; algorithm created via expert consensus + empirical data	Research tool (Delphi-based algorithm; not intended for PHC)	Delphi-derived clinical and radiological criteria	Culture-confirmed TB	Sensitivity 88%; Specificity 96%

AFB – acid-fast bacilli, CXR – chest x-ray, HIV – human immunodeficiency virus, NTLP – National Tuberculosis and Leprosy Programme, PHC – primary health care, PTB – pulmonary tuberculosis, SNPTB – smear-negative pulmonary tuberculosis, TB – tuberculosis, TST – tuberculin skin test, TPS – tuberculosis prediction score, WHO – World Health Organization

*Country-specific vs research tool algorithm

†Sensitivity/specificity

**Table 2:** AI-generated summary of classical and AI-enhanced diagnostic algorithms for smear-negative pulmonary tuberculosis (SNPTB)

Study, year	Country	Population	Type of algorithm*	Algorithm used	Comparator†	Performance‡
Chierakul *et al*., 2016 [18]	Thailand	Adults investigated for smear-negative PTB	Research tool	Symptom- and CXR-based radiographic feature algorithm	Not explicitly compared to WHO; evaluated *vs*. TB diagnosis	Feature-level CXR sensitivity/specificity = 37–80%
Nakiyingi *et al*., 2021 [19]	Uganda	HIV-TB co-infected adults with smear-negative disease	Research tool	CXR interpretation alone *vs*. CXR + Xpert	Xpert MTB/RIF	CXR sensitivity = 67.9%; CXR + Xpert 87.7%
Souza Filho *et al*., 2016 [20]	Brazil (Rio de Janeiro)	Sputum smear-negative TB suspects in general hospital setting	Research tool (AI/ANN model)	ANN using 12 clinical variables	Internal data set; compared with clinical diagnosis	Sensitivity = 78–100%; specificity = 54–93%
Kazemzadeh *et al*., 2024 [21]	Zambia (Lusaka)	Prospective multi-site programme screening population	Research tool (AI-CAD; programmatic evaluation)	DL CAD for CXR	Radiologists & WHO triage thresholds	AUC = 0.87; tunable sensitivity/specificity meeting WHO triage targets
Chung *et al*., 2024 [22]	South Korea	Mobile-unit population screening for TB	Research tool (AI-CAD)	DL CAD for infectivity prediction on mobile CXR	Radiologist interpretation	AUC = 0.95; sensitivity = 94.7%; specificity = 83.3%; ~ 60% radiologist workload reduction
Huang *et al*., 2022 [23]	Taiwan (Huang)	Multi-centre smear microscopy patients	Research tool (AI-automated microscopy)	μ-Scan 2.0 automated AFB detection	Human microscopy readers	Sensitivity = 88–92%; specificity = 96–98%; higher yield *vs*. manual smear
Chen *et al*., 2024 [24]	Taiwan (Chen)	Patients undergoing smear microscopy for TB	Research tool (AI/automated AFB scanner)	Intelligent microscopy scanner + image recognition	Manual smear microscopy	Sensitivity = 88–92%; specificity = 96–98%
Zhao *et al*., 2024 [25]	China	Smear-negative TB suspects (sputum & BALF samples)	Research tool (molecular assay)	SAT-TB rapid RNA amplification	Xpert MTB/RIF (when combined)	SAT-TB alone: moderate sensitivity, perfect specificity; SAT-TB + Xpert: overall sensitivity ~ 70%
Codlin *et al*., 2024 [26]	Multi-country (Bangladesh, Pakistan, Zambia, Vietnam)	Modelling of high-burden TB programme settings	Research tool (AI-assisted pooled testing)	AI-guided pooled Xpert testing	Standard individual-sample Xpert testing	Similar diagnostic yield; ~ 50% reduction in cartridge use
Bigio *et al*., 2021 [27]	Global (systematic review)	Adults undergoing lung ultrasound (LUS) for TB triage	Research evidence synthesis	Point-of-care lung ultrasound for subpleural nodules	Not compared to WHO algorithm; pooled diagnostic accuracy	Sensitivity = 72.5–100%; limited specificity; evidence heterogeneous
Suttels, 2024 (PhD dissertation) [28]	DR Congo/Africa (TrUST/ULTR-AI)	Adults in TB-endemic regions using smartphone LUS	Research tool (AI-LUS)	ULTR-AI automated lung ultrasound triage suite	WHO triage target thresholds	Meets WHO triage criteria: sensitivity ≥90%; specificity ≥70%

AFB – acid-fast bacilli, AI – artificial intelligence, ANN – artificial neural network, AUC – area under the receiver operating characteristic curve, BALF – bronchoalveolar lavage fluid, CAD – computer-aided detection, CXR – chest x-ray, DL – deep learning, HIV – human immunodeficiency virus, LUS – lung ultrasound, ML – machine learning, NTLP – National Tuberculosis and Leprosy Programme, PHC – primary health care, PTB – pulmonary tuberculosis, SAT-TB – simultaneous amplification test for tuberculosis, SNPTB – smear-negative pulmonary tuberculosis, TPS – tuberculosis prediction score, TST – tuberculin skin test, WHO – World Health Organization, Xpert – xpert MTB/RIF molecular assay

*Country-level vs research tool algorithm

†Examples of comparators: WHO algorithm, radiologist, Xpert MTB/RIF, and expert panels.

‡Sensitivity/specificity/AUC.

However, these more advanced diagnostic tests are not widely accessible in Sabah. Given the limitations of existing algorithms and the unavailability of alternative, higher-cost diagnostic tools, there is a need for a practical clinical algorithm to support the diagnosis of SNPTB among symptomatic patients in settings without access to molecular testing or robust laboratory infrastructure. Therefore, we aimed to develop a locally relevant clinical algorithm scoring system by conducting a modified Delphi exercise to obtain consensus from a panel of experts.

## METHODS

### Study design: the modified Delphi method

The Delphi method and its modifications have been widely used in health sciences for obtaining a reliable consensus from a group of experts on a specified area [29]. It normally involves several rounds of surveys in which people vote until consensus is reached. The Delphi method can be modified, for example, by including focus group discussions, so long as it leads towards a group consensus.

For this study, we modified the Delphi method by using an online survey platform to enable as many experts as possible to participate. Care was taken to ensure the responses from the experts were collated and de-identified after each round to reduce the dominance of one expert as well as to avoid groupthink. We conducted three rounds of online surveys, after which we held a hybrid consensus meeting to further refine and apply weightings to the parameters in the algorithm.

### Study participants

There is no established method to determine the sample size needed for a Delphi study, as it often depends on the topic area and convenience; while a sample of <10 participants is considered insufficient, a very high number would not necessarily improve the quality of the results. Akins *et al*. [30] recommended the use of at least 23 panellists to ensure reliability.

Here, we identified 27 experts from within Malaysia and internationally and invited them to participate. Individuals had to be TB experts with a minimum of five years’ experience in the management of TB and had to be representative in the management and diagnosis of SNPTB in Sabah. Experts were identified through consultation with local TB stakeholders and by reviewing the list of authors of the latest national clinical guidelines on TB management. Their contact details were obtained from their organisations’ webpages, and they were initially contacted via email or telephone. One international expert accepted the invitation, but subsequently did not respond-when the consent form was sent despite reminders.

### Round 1, 2, and 3 survey: questionnaire and analysis

Round 1 of this Delphi survey, conducted in January 2024, aimed to generate a list of parameters for diagnosing TB among symptomatic SNPTB patients in Sabah. The experts were asked regarding their role and experience in managing patients with TB, followed by open-ended questions ‘Q1: what parameter(s) do you think should be included in an algorithm to diagnose smear-negative PTB?’ and ‘Q2: Can you suggest any recent studies or publications that may be relevant to this study? We analysed the answers to Q1 thematically, grouping the suggested parameters into themes and forming a preliminary framework. We also reviewed the publications suggested in Q2 and incorporated the identified parameters into the preliminary framework. After this round, we listed all identified parameters and removed duplicates.

The round 2 survey aimed to distil the proposed list of parameters to diagnose SNPTB. Specifically, we sent the preliminary framework back to the group of experts and asked them to rate the importance of each parameter identified in diagnosing SNPTB in primary care using a five-point Likert scale (1 = ‘not important at all’; 2 = ‘slightly important’; 3 = ‘moderately important’; 4 = ‘very important’; and 5 = ‘extremely important’. An *a priori* criteria for consensus required ≥70% of the respondents to rate of an item as ‘very important’ or ‘extremely important’ to be included in the refined framework.

The round 3 survey aimed to further refine the framework and attempted to assign a weight to each parameter. We sent this refined framework to the group of experts and asked them to score each item on its likelihood of being a marker for smear negative pulmonary TB using a scale from 0 (unlikely) to 100 (likely).

We collected data using the REDCap secure online platform for all the rounds. New questionnaires were formulated for the subsequent rounds based on the preceding round until a consensus was reached. Each round of questionnaires took approximately 30 minutes to complete, and the experts were given up to three weeks to respond, with reminders sent each week.

### Hybrid expert consensus meeting

We initially intended to use the mean score for each parameter at round 3 as the weighting of the parameter. However, we found that the mean scores of the parameters were not distinctive, thus a consensus meeting of experts was convened, where each parameter was discussed and voted on to decide on the inclusion as well as weighting of each parameter (from 0 to 10) in the algorithm. In the event of disagreement, further discussions were carried out, and the final consensus was obtained using a simple majority by voting among those present.

### Cut-off score of the clinical algorithm

We used a pragmatic approach to obtain a preliminary cut-off score for ‘likely smear negative TB’ *vs*. ‘unlikely smear negative TB’ by using existing (retrospective) secondary data set of 60 patients from a university hospital. Twenty-nine (48.3%) were confirmed as having smear negative TB using MTB culture, while 31 (51.6%) were not.

We applied the algorithm (including parameter weightings) to both the negative and positive cases, calculated their mean scores with 95% confidence intervals (CIs), and performed an independent samples *t*-test to determine any differences between the two. Additionally, we ran a receiver operating curve (ROC) analysis to determine the overall discriminative power based on the area under the curve (AUC) and its 95% CI. We then referred to a coordinates of the curve table to identify the optimal cut-off point based on Youden Index and sensitivity value [31]. Lastly, we calculated the sensitivity, specificity, positive predictive value (PPV), and negative predictive value (NPV) for the selected cut-off point.

We performed all statistical analysis in SPSS, version 27 (IBM Corp., Armonk, NY, USA).

### Final review and agreement of the clinical algorithm

We circulated the draft version of the weighted clinical algorithm with the cut-off point to the experts via email, presenting the process of how the final algorithm was established, as well as the sensitivity, specificity, PPVs, and PNVs. We then asked participants whether they agreed with the final algorithm and invited further comments, which we then reviewed and incorporated, if relevant, into the final version of the algorithm.

## RESULTS

**Figure 1:**
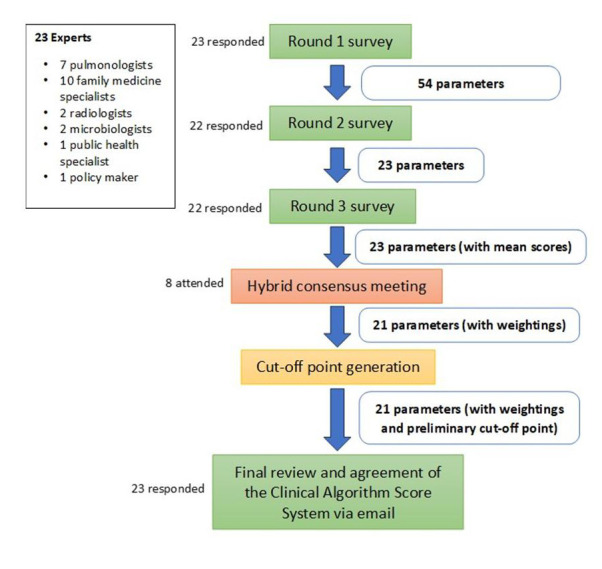
Flow diagram of expert participation in the modified Delphi process.

Twenty-three (85.2% of the 27 identified and invited experts consented to participate (**Figure 1**), all of whom were from Malaysia. Twenty-two (95.6%) participated in all the rounds, and one dropped out after round 1. Only eight experts (34.8%) comprising pulmonologists, family medicine specialists, and a policymaker attended the hybrid consensus meeting. Two specialties (radiology and microbiology) were not represented at this meeting. We emailed the algorithm with the weightings and the computation method to all remaining 15 experts to elicit their inputs. All replied promptly with no additional comments, and all agreed unanimously to the final algorithm without any further changes.

**Figure 2:**
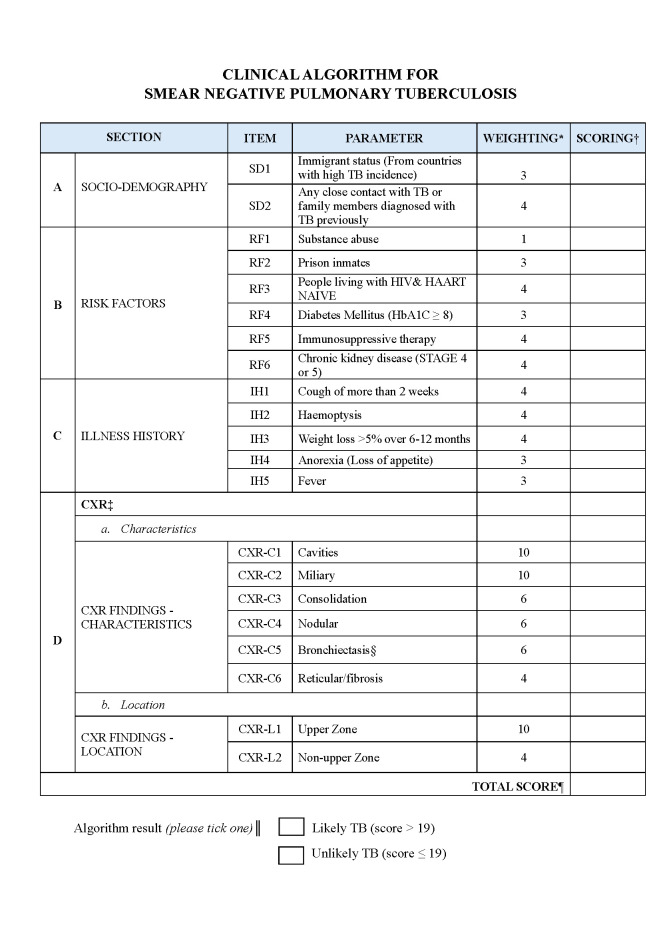
The final clinical algorithm for SNPTB with scoring system. This figure illustrates a weighted, point-based clinical scoring algorithm developed to aid the diagnosis of SNPTB in symptomatic patients with negative sputum smear microscopy. The algorithm is intended for use in resource-limited settings to support clinical decision-making when rapid molecular diagnostics are unavailable. It integrates four domains: socio-demographic factors (**Panel A**), clinical risk factors (**Panel B**), illness history (**Panel C**), and chest radiograph (CXR) findings (**Panel D**), including both radiographic characteristics and anatomical location. *Each parameter is assigned a predefined weighting based on expert consensus. †Each parameter is scored independently. ‡For CXR, the scores can be more than one according to the presence of the described lesion(s). §CXR with bronchiectasis features will require further clinical work-up. ¶The total score is the sum of the total weightings. ║A total score >19 indicates likely TB, while a score ≤19 indicates unlikely TB. The scoring should take only 2–5 minutes to complete. CXR – chest x-ray, IH – illness history, RF -risk factors, SD – sociodemographic.

We identified fifty-four parameters from round 1, categorising them by ‘sociodemographic backgrounds’, ‘risk factors’, ‘illness history’, ‘physical examination’, ‘laboratory investigations’, and ‘radiological investigations’ (**Online Supplementary Document**). After round 2, only 23 parameters were retained. All parameters under ‘laboratory investigations’ were dropped. These 23 parameters were then scored (0–100) in round 3; the mean score of each parameter ranged from 61.5 to 88.1, while the overall mean score across all parameters was 75.3 (standard deviation = 7.95), indicating moderate variability in parameter weightings The mean scores of the parameters were not distinctive, including the CXR parameters, which were hypothesised to have a higher weighting in predicting SNPTB. Seven out of the 23 parameters were dropped in the hybrid consensus meeting, but five new ones were added under the CXR category to improve clarity. This led to a final algorithm of 21 parameters with the categories ‘sociodemographic background’, ‘risk factors’, ‘illness history’, and ‘CXR characteristics and location’. The weightings allocated for each parameter ranged from 1 to 10 (**Figure 2**).

**Figure 3:**
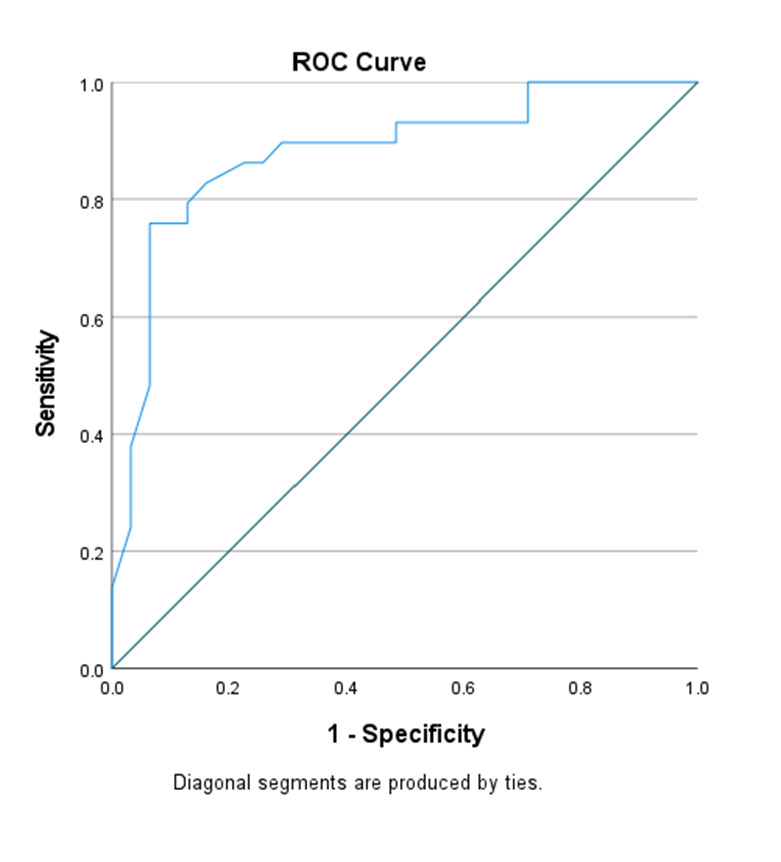
ROC curve of the clinical algorithm generated based on the 60 patient records. The ROC curve illustrates the discriminative performance of the weighted clinical algorithm applied to 60 patient records, comparing smear-negative pulmonary tuberculosis cases with non-TB controls. The AUC was 0.88 (95% CI = 0.79–0.97), indicating good diagnostic accuracy. ROC – receiver operating characteristic.

After applying the parameter weightage to the 60 patients’ records, the mean total score was 11.8 (95% CI = 9.6–14.0) among negative cases and 28.4 (95% CI = 26.6–30.2) among positive cases. An independent samples *t* test demonstrated a difference in mean scores between the two groups (*P* < 0.001), while the ROC analysis indicate the clinical algorithm achieved an AUC of 0.88 (95% CI = 0.79–0.97) (**Figure 3**). Based on the coordinates of the curve table, a cut-off score of 19.5 yielded a Youden index of 0.636, sensitivity of 86.2%, specificity of 77.4%, PPV of 78.1%, and NPV of 85.7% (**Table 3**). All experts (100%) agreed to this version of the clinical algorithm-based scoring system and the proposed cut-off score of 19.5.

**Table 3:** Confusion matrix for the 60 patients’ records using the cut-off score of 19.5*

		Actual diagnosis		
		**Positive**	**Negative**	
**Diagnosis based on 19.5 cut-off score**	**Positive**	25 (true positive)	7 (false positive)	PPV = 25/32 (78.1%)
	**Negative**	4 (false negative)	24 (true negative)	NPV = 24/28 (85.7%)
		Sensitivity = 25/29 (86.2%)	Specificity = 24/31 (77.4%)	

NPV – negative predictive values, PPV – positive predictive value

*This table summarises the diagnostic performance of the clinical algorithm in 60 patients with suspected smear-negative pulmonary tuberculosis, using a cut-off score of 19.5 to classify cases as TB-positive or TB-negative. The matrix shows the distribution of true positives, false positives, false negatives, and true negatives in comparison with the reference diagnosis. At this threshold, the algorithm demonstrated a sensitivity of 86.2%, specificity of 77.4%, PPV of 78.1%, and NPV of 85.7%, indicating good overall discriminatory ability in this cohort.

## DISCUSSION

In this modified Delphi study, we developed a 21-parameter clinical algorithm to diagnose SNPTB in Sabah state, Malaysia. This algorithm included sociodemographic, risk factor, illness history, and CXR parameters, achieved an AUC of 0.88, and, based on a cut-off score of 19.5, yielded a sensitivity of 86.2%, specificity of 77.4%, PPV of 78.1%, and NPV of 85.7%. However, this preliminary cut-off point may change pending the results of a larger prospective validation study to be carried out in Sabah.

Our initial inclusion of three sociodemographic factors after round 3 (immigrant status, close contact with a TB patient, and overcrowding) is consistent with existing literature, in which many sociodemographic characteristics have been associated with developing TB [32–36]. However, many of these studies were country. or context-specific [34–36], and while overcrowding has been cited in some literature as a risk factor for TB transmission in Malaysia [34,37], it was either linked to institutional settings like prisons or poor socioeconomic factors such as poverty and low-cost housing. There are, to our knowledge, no local studies that quantify number of persons per room in relation to overcrowding and TB infection. Overcrowding was dropped as a parameter in our consensus meeting, as the panel decided that its importance was related to ventilation, which is not an important factor in Malaysia in view of the hot tropical climate that necessitates most houses keeping the windows open. Other associated factors, such as being in institutions like prisons, had already been accounted for in the algorithm.

Six symptoms identified after round 3 were cough duration more than two weeks, haemoptysis, weight loss >5% over 6–12 months, anorexia, fever, and productive cough. The panel decided to drop productive cough, consistent with the list suggested by the WHO [38].

CXR has been shown to improve the yield of diagnosis of TB among smear-negative patients, although its sensitivity and specificity by itself is inadequate [39]. The panel agreed that there are two important components in the CXR manifestations that are suggestive of TB, *i.e.* both the appearance and the location of the lesions, which was supported by literature [40]. It was decided that weightings should be applied separately for these two factors.

Our clinical algorithm was generated for a high TB incidence, but low HIV prevalence setting. We are now validating this algorithm in a prospective study in our setting. However, even when our full validation study is complete, our algorithm will not be appropriate for use in countries with high HIV setting prevalence, as the CXR findings in HIV patients are likely to be atypical.

Our study has produced a context-specific algorithm for the diagnosis of SNPTB in Sabah, based on the knowledge of Malaysian experts. If future validation demonstrates that this algorithm is non-inferior to rapid molecular diagnostic tests, it could contribute to identifying the ‘missing millions’ of people with TB in Sabah, Malaysia. While the presence of experts who developed the other existing algorithms in low- and middle-income countries may have been beneficial, our algorithm was created for Sabah and thus may not be generalisable to other such context, like those with a high HIV prevalence. The low participation of the experts, along with the absence of radiologist and microbiologist in the consensus hybrid meeting, could have affected the robustness of the algorithm. Lastly, we used a data set of 60 individuals in an urban university hospital for the provisional algorithm (before the full validation study), which may have limited generalisability.

The developed clinical algorithm is intended for use by doctors in primary care settings, particularly in resource-limited areas with restricted access to GeneXpert facilities. It is easy to use and only takes 2–5 minutes to complete. As CXR is a major component of this algorithm, it is important that to ensure its interpretations by primary care doctors are accurate, as radiologist review is not feasible due to limited human resources. Regular training workshops and periodic audits should thus be conducted to enhance the reliability and consistency of CXR interpretation.

## CONCLUSIONS

In our preliminary study, we present a clinical algorithm to diagnose SNPTB in resource-constrained settings that have no or limited access to GeneXpert and culture. The algorithm is currently being validated in a larger study.

## Additional material


Online Supplementary Document
