# Supplementary material for: Development of a clinical algorithm-based scoring system to diagnose smear-negative pulmonary tuberculosis in Sabah, Malaysia using the modified Delphi method
Source: J Glob Health. 2026 Feb 20;16:04085. doi: 10.7189/jogh.16.04085 (PMC13002176; doi:10.7189/jogh.16.04085)
Supplement: Online Supplementary Document [file jogh-16-04085-s001.pdf]

Supplement to: Wong CK, Lee WK, Teo R, Ramamurthy HY, Dony J, Teo CH, Chan SJJC, Sivasegaran S, Lew YL, Lam RH, Chinna K, Rajahram GS, William T, Chan YC, Jeevajothi Nathan J, Nair H, Campbell H, Khoo EM, Stagg HR; AMASSMENT Expert Panel, RESPIRE Collaboration. Development of a clinical algorithm-based scoring system to diagnose smear-negative pulmonary tuberculosis in Sabah, Malaysia using the modified Delphi method. J Glob Health. 2026;16:04085.

Appendix 1

List of parameters Round 1

Sociodemographic background

- Age
- Gender
- Country of origin
- Immigrant status
- Ethnic group
- Any close contact with TB or family members diagnosed with TB previously
- Occupation
- Poverty
- Overcrowding
- Smoking/vaping history

Risk factors

- Substance abuse
- Prison inmates
- People living with HIV
- Alcohol abuse
- Diabetes mellitus
- Immunosuppressive therapy
- Chronic kidney disease

Patient's illness history

- Chronic cough of more than 2 weeks
- Haemoptysis
- Weight loss
- Anorexia
- Asthenia/fatigue
- Lethargy
- Fever
- Night sweats
- Shortness of breath (SOB)
- Productive cough

Physical examination

- Compatible with extrapulmonary TB e.g. TB Meningitis
- Cervical lymphadenopathy
- Pallor

- Dry Skin
- Cachexic
- Thin, body habitus
- Low BMI (<18.5)
- Evidence of weight loss (documented weight loss)
- Blood pressure
- Pulse rate
- Respiratory rate
- Oxygen saturation on air (SpO2)
- Elevated capillary glucose
- Abnormal respiratory examination (e.g. reduced air entry, crepitation
- BCG scar

Laboratory investigations

- Full blood count (FBC)
- Erythrocyte sedimentation rate (ESR)
- HIV serology
- HbA1c
- Sputum centrifugation for AFB stain to increase yield

Radiological investigations and findings

- Chest X-ray (CXR)
- CXR with artificial intelligence

CXR findings

- Cavities or consolidation in upper lobe zones
- Hilar adenopathy
- Apical infiltrates
- Pleural effusion
- Any parenchymal lesions at any site (not necessarily upper zone opacities) because smear negative PTB normally presented with atypical CXR findings
